# English and Foreign Spas

**Published:** 1899-11-18

**Authors:** 


### ENGLISH AND FOREIGN SPAS.

England possesses many spas, but it may be questioned whether English people—even those resident at and interested in the spas themselves—take the treatment there given seriously, or regard it, as is done abroad, as an all-important rite. A correspondent, writing in the *Times* a few weeks ago, in praise of Strathpeffer, describes the Nauheim treatment as carried out there; and, while pointing out the advantages of the shorter and more comfortable journey, of the good hotels, and of the invigorating air, goes on to say: "But there is the *revers de la médaille*." In Nauheim everything is done with a view to assure the success of the heart cure. The bath houses are perfect, the baths being all on the ground floor, and partly sunk into the floor, so that there is no effort either in getting into or out of them, while the attendance is excellent. At Strathpeffer, on the other hand, he says: "The Nauheim baths are up a steep flight of steps, and constructed of such slippery marble that to get in and out of them is a feat of gymnastics . . . which would be dangerous to anyone in an advanced state of heart disease." Again, he compares the position of the heart patient at the two places. "In Nauheim the heart patient is all-important. In Strathpeffer he is a mere casual. In Nauheim the doctor sees him every day, and advises as regards the next day's bath, and a moderate fee at the close of the cure covers all such attendance. In Strathpeffer the Nauheim patient counts for very little, and if he . . . desires advice as to his fitness for each succeeding bath, he must specially seek out his doctor each day." Of course, this is to a certain extent a grumble; still it hits the nail fairly on the head, for it is certainly the fact that, not in regard to one spa in particular, but as a general thing, the link between the doctor and the bath establishment is much less complete in England than in some places abroad, and we cannot but believe that the English bathing places suffer in consequence. It is a matter of great public importance that English bathing places should be utilised to the full. There is no doubt about their efficacy, but "the authorities . . . must set their baths in order, and make them as comfortable as those of Nauheim, and the doctors must contrive to give more individual attention to their patients if they are really in earnest in their rivalry" with foreign spas. So says the patient even in the midst of his gratitude for good received, and what he says may well be taken to heart by the authorities at many watering places. If the full advantage of bathing is to be obtained, the patient must be under constant medical supervision.

